# A Pathogenetic Materia Medica

**Published:** 1895-09

**Authors:** 


A Pathogenetic Materia Medica: Based upon Drs. Hughes' and Dake's Cyclopædia of Drug Pathogenesy by the Medical Investigation Club of Baltimore, Maryland. Philadelphia, Pa.: Bœricke & Tafel, 1895. Price, cloth, $2.00; net by mail, $2.17.

The Medical Investigation Club of Baltimore is an organization composed of the following members, all of whom are professors in the Southern Homœopathic Medical College: Dr. Elias C. Price, Professor Institutes and Hygiene; Dr. Eldridge C. Price, Professor Materia Medica and Therapeutics; Dr. Robert W. Mifflin, Professor Pathology and Practice; Dr. O Edward Janney, Professor Pædology and Orthopædic Surgery; Dr. George T. Shower, Associate Professor Physiology, Pharmacy, and Toxicology, and Dr. Henry Chandlee, Professor of Obstetrics. All these gentlemen are associated together with the avowed object of advancing the cause of Homœopathy by settling the question of the authenticity of the provings made and recorded in the accepted works of materia medica in the homœopathic school. This highly praiseworthy intention they seek to attain by conducting provings in themselves and others; by ransacking the literature of poisons for the most authentic symptoms recorded of poisoning cases; by re-examining the daybook records of provers of medicines for the homœopathic school, and by drawing upon the *Cyclopœdia of Drug Pathogenesy*, Dr. Allen's *Encyclopedia*, *Metcalf's Provings*, and the *Transactions of the American Institute.*

In conducting such a laborious task they have thrown out all clinical symptoms, all keynotes, and other symptoms not observed on at least ten persons.

The constant repetition of these observed symptoms in different people is deemed absolutely essential to enable them to declare the symptoms authentic and reliable.

This plan is, of course, directly in the line of, as well as harmonious with, the methods in use in every physical laboratory, by every original investigator, who tests his experimental results repeatedly under all sorts of varied conditions before he arrives at his conclusions.

It appeals to the sympathy of every scientific intellect, and seems as if it must produce only the highest results, and as a matter of fact their work has been cordially received by the profession through the medium of the two journals in which it has been published from time to time. This encouragement has inspired them to incorporate it in a book. That book is now before us, and is the subject of this review.

In looking over the work we are at once struck with the reduction in the number of symptoms. The pathogenetic record of each of these drugs is remarkably reduced. Many of our most valued "indications" are not present, while on the other hand many other familiar ones are quite conspicuous.

While it is desirable that the volume of our materia medica should be reduced, and while it is apparent that much of it is erroneous and untrustworthy, it is very doubtful if it can be reformed by wholesale cutting out of large numbers of symptoms by the setting up of an arbitrary standard which, while it rules out much that is worthless, also cuts down the old established symptoms on which we are accustomed to rely.

In other scientific studies, correction of error is not brought about by any kind of arbitrary cutting out of results presumed, *à priori*, to be untrue, but by the laborious testing over again experimentally, of each step in the process by which the result was obtained. It would seem that such must be the method in the materia medica of the new school. The determining the value of a symptom is not to be reached by reasoning, but by testing the drug in the shape of a proving upon people who are not sick, and by observing the effects of applying it clinically in sick conditions. This is necessarily a slow, step-by-step process, which, however, will be effectual if done with sufficient accuracy, patience, and industry. It *is* being done by lots of practitioners. *All* who laboriously study out a close comparison of the symptomatology of a case with the recorded symptomatology of the materia medica, who give the remedy which seems to be truly indicated, who record the results of treatment carefully, and then send the records to a reliable homœopathic journal for publication, all these men are contributing to the solution of the problem as to the truth or falsehood of the symptoms of the materia medica. It is therefore the duty of us all to record and publish every case we treat, whether successful in curing or not, for the sake of contributing to this record. This, we think, is the *true* way of reforming the materia medica. It is the scientific way, it is modeled after the methods of investigation followed in other fields of scientific inquiry, and it takes away the loss that comes to any built-up system which has been the work of a particular discoverer, when it is subjected to the scrutiny of arbitrary minds governed by *à priori* opinions, who are necessarily tyrranical and unconsciously unjust in their judgments.

This same method of reforming the materia medica, open to the same objections as here stated, was made some years ago by the builders of the *Encyclopedia of Drug Pathogenesy*. They produced a work which, however earnest and honest their efforts, has no place in the practical, every-day work of the active practitioner.

In the work now under review, we do not perceive much practical testing of remedies upon provers; it seems rather to be a literary research into the various symptomatologies, and records of provers, that have already been published. This can hardly be called original work, which is what is wanted in such an undertaking as this book represents.

A mistaken motive also has influenced the investigators which actually handicaps their otherwise admirable desire to reform and render indisputable the homœopathic materia medica.

That handicapping motive can be found in the following quotation from the introduction at page xvi:

"The endeavor of the most progressive members of the older school of medicine of late years has been to study what they term physiological drug effects. These physiological effects are none other than what may be more correctly called pathogenetic effects. Consequently, as this volume deals exclusively with pathogenetic drug effects, the attention of the student of the older theories of therapeutic application may be attracted to it, and as no other work exists in which pure and exclusive pathogenetic effects are placed in relation to homœopathic therapeutics, it is to be hoped that this effort will initiate a desire among our brethren of the older school to drop all sectarian limitations, and, with a determination to apply only the strict impartial tests of science to the analysis of Homœopathy, make a critical study of the relation of pathogenesy to pathology."

Here we have exhibited a desire to disarm the hostility of the old school; an attempt to placate their malignity; a wish for union with them on terms of equality. Vulgarly speaking, it is throwing a tub to the allopathic whale! This is the motive that handicaps the work of these gentlemen. It is the motive that has largely influenced the builders of *The Cyclopedia of Pathogensy*. It is the motive that actuates a large majority of the professed adherents of our school who come into it, reject it, and then practice a mixed new school and old-school therapeutics, variously denominated eclecticism, mongrelism, liberalism, rationalism, etc., while they fill the journals and society proceedings with revelations of their scepticism of the system they profess.

This does not remove the resistance of the old school at all. They are just as earnest and probably as honest in their antagonism as we are in our devotion to the Hahnemann system. They will not be converted in this way. Consequently, all these efforts to captivate them in the ways here indicated are so much wasted energy.

There is no intention here to make any unkind or hostile criticism of the work of these reformers as shown forth in this volume; but we find it handicapped, as before stated, by this desire to cater to the other school, and that will nullify it. It has made the *Cyclopedia* a failure because it has made it useless, and the anxious prescriber is unable to find the simillimum from a perusal of its pages.

Any author who seeks to deprive the homœopathic physician of such indicating symptoms as the aggravation from jarring of Belladonna, the perspiration during sleep of Calcarea, the flushes of heat of Sulphur, or any other trusted and tried symptom of the materia medica upon which he relies to relieve his cases of sickness, is likely to receive a very cold reception from the medical worker to whom he appeals.

